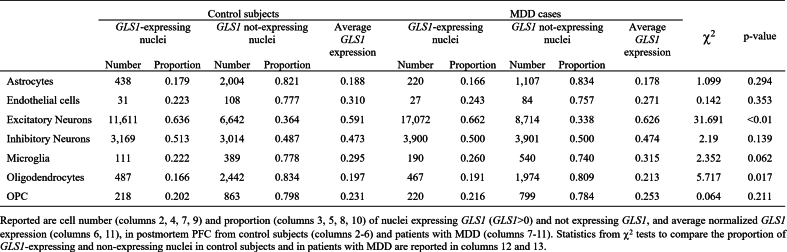# Inhibition of microglial glutaminase alleviates chronic stress-induced neurobehavioral and cognitive deficits

**DOI:** 10.1016/j.neurot.2025.e00759

**Published:** 2025-09-27

**Authors:** Meixiang Huang, Yannan Li, Ajit G. Thomas, Anjali Sharma, Wathsala Liyanage, Tomáš Tichý, Lukáš Tenora, Yu Su, Jisu Ha, Niyada Hin, Mizuho Obayashi, Pavel Majer, Rangaramanujam M. Kannan, Takashi Tsukamoto, Gianluca Ursini, Rana Rais, Barbara S. Slusher, Xiaolei Zhu

**Affiliations:** a Johns Hopkins Drug Discovery, Johns Hopkins University School of Medicine, Baltimore, MD 21205, USA; b Department of Neurology, Johns Hopkins University School of Medicine, Baltimore, MD 21205, USA; c Department of Psychiatry and Behavioral Sciences, Johns Hopkins University School of Medicine, Baltimore, MD 21205, USA; d Center for Nanomedicine, Department of Ophthalmology, Wilmer Eye Institute, Johns Hopkins University School of Medicine, Baltimore, MD 21231, USA; e Institute of Organic Chemistry and Biochemistry, Academy of Sciences of the Czech Republic (ASCR), Prague 160 00, Czech Republic; f Lieber Institute for Brain Development, Johns Hopkins Medical Campus, Baltimore, MD 21205, USA; g Department of Chemical and Biomolecular Engineering, Johns Hopkins University School of Medicine, Baltimore, MD 21205, USA; h Department of Physiology, Pharmacology & Therapeutics, Johns Hopkins University School of Medicine, Baltimore, MD 21205, USA; i Department of Neuroscience, Johns Hopkins University School of Medicine, Baltimore, MD 21205, USA; j Department of Oncology, Johns Hopkins University School of Medicine, Baltimore, MD 21205, USA; k Department of Medicine, Johns Hopkins University School of Medicine, Baltimore, MD 21205, USA

**Keywords:** Microglia, Glutaminase, Chronic social defeat stress, Dendrimer, Depression

## Abstract

Major depressive disorder (MDD) is a prevalent and debilitating psychiatric condition with significant societal and economic impacts. Many patients are resistant to current antidepressant therapies, underscoring the need for novel treatments targeting underlying mechanisms. We previously discovered that glutaminase (GLS1), an enzyme converting glutamine to glutamate, is upregulated specifically in activated microglia in mice exposed to Chronic Social Defeat Stress (CSDS). Importantly, GLS1 mRNA was also upregulated in microglia within postmortem brain tissue of MDD patients, highlighting a potential role for microglial GLS1 in MDD pathophysiology. However, existing GLS1 inhibitors lack brain penetrance and/or cause gastrointestinal toxicities, limiting their translational potential. To address this, we utilized a hydroxyl-terminated poly(amidoamine) dendrimer nanoparticle system to selectively target microglial GLS1. Using structurally distinct GLS1 inhibitors, we synthesized two hydroxyl-dendrimer-GLS1 inhibitor conjugates: dendrimer-TTM020 (D-TTM020) and dendrimer-JHU29 (D-JHU29). In the murine CSDS model, we evaluated their microglial target engagement, safety, and efficacy using immunofluorescence, GLS1 activity assays, gastrointestinal histopathology, and a battery of behavioral tests. Using a Cy5 fluorescently labeled hydroxyl-dendrimer (D-Cy5), we confirmed that systemically administered D-Cy5 crossed the blood-brain barrier and was selectively engulfed by activated microglia in mice after CSDS. D-TTM020 and D-JHU29 attenuated CSDS-induced microglial GLS1 activity elevation without affecting non-microglial cells. Furthermore, D-TTM020 and D-JHU29 both alleviated CSDS-induced social avoidance, and D-TTM020 additionally reduced anxiety-like behavior and improved recognition memory. Both conjugates were well tolerated, with no overt or gastrointestinal toxicities. Collectively, these findings suggest that microglia-targeted GLS1 inhibition is a promising therapeutic approach for chronic stress-associated depression.

## Introduction

Major depressive disorder (MDD) is one of the most prevalent psychiatric disorders, with nearly one in five Americans affected during their lifetime [1,2]. Despite the availability of effective treatments such as selective serotonin reuptake inhibitors (SSRIs) and serotonin-norepinephrine reuptake inhibitors (SNRIs), approximately 30–50 ​% of patients exhibit treatment resistance [[3], [4], [5], [6], [7], [8]]. This highlights a critical gap in addressing the underlying pathophysiological mechanisms of MDD and underscores the necessity for novel pharmacological agents targeting different mechanisms [3].

Emerging evidence suggests a significant role for glutamate signaling in MDD [[9], [10], [11]]. Patients with MDD exhibit elevated blood glutamate levels, increased brain concentrations of glutamine —the primary precursor for glutamate—and higher glutamine-glutamate ratios in cerebrospinal fluid (CSF) [[12], [13], [14], [15]]. These findings are supported by recent studies demonstrating increased or altered glutamate content in the brains of MDD patients [[16], [17], [18]]. Additionally, the ionotropic N-methyl-d-aspartate (NMDA) glutamate receptor antagonist, ketamine, and modulators of metabotropic glutamate receptors have gained greater attention as novel antidepressant agents [[18], [19], [20], [21], [22]]. Ketamine induces rapid and sustained antidepressant effects in treatment-resistant patients [[23], [24], [25]], with positive responses linked to alterations in extracellular glutamate availability and signaling [[18], [19], [20], [21], [22]]. Collectively, these findings suggest that modulating glutamate pathways could be a promising strategy for treating MDD.

Along with glutamatergic dysfunction, neuroinflammation has been increasingly recognized as a critical contributor to MDD pathophysiology [[26], [27], [28]]. Clinical evidence has shown elevated levels of inflammatory markers in the blood, CSF, and brain tissue of MDD patients [[26], [27], [28]]. Microglia, the resident immune cells of the central nervous system (CNS), play a central role in neuroinflammation and glutamate regulation [29,30]. Activated microglia release excessive amounts of glutamate through the upregulation of glutaminase 1 (GLS1, official gene symbol: GLS), an enzyme that catalyzes the conversion of glutamine to glutamate [[31], [32], [33]]. This aberrant glutamate production leads to excitotoxicity and neuronal damage, which are key mechanisms implicated in the development of depressive symptoms [[26], [27], [28],^34^,^35^].

**Fig. 1:**
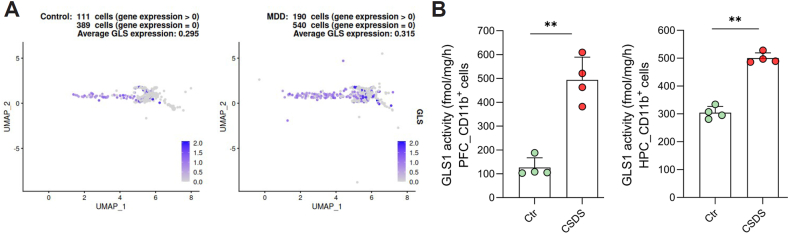
**Elevated microglial GLS1 mRNA and enzymatic activity in post-mortem MDD cases and mouse brain following CSDS, respectively.** (**A**) The Uniform Manifold Approximation and Projection (UMAP) plot colored by velvet revealed a higher proportion of microglia cells expressing *GLS1* in MDD cases vs. control subjects, as supported by a strong trend (111/500 vs. 190/730, Chi-squared ​= ​2.352, *p* ​= ​0.062). Control subjects, n ​= ​17, MDD cases, n ​= ​17. Data were analyzed by χ^2^ test. See Supplementary Table 1 for further details. (**B**) GLS1 enzyme activity was measured in microglia-enriched CD11b^+^ cells isolated from the PFC and HPC of CSDS mice and control mice using a GLS1-specific assay, with results expressed as fmol/mg/h. GLS1 activity in microglial cells was significantly higher in CSDS mice compared to controls. Data were presented as mean ​± ​SEM, Control, n ​= ​4; CSDS, n ​= ​4. Data were analyzed using an unpaired Student's *t*-test. ∗∗*p* ​< ​0.01.

Given the prominent role of microglia and GLS1 in the production of excitotoxic glutamate, GLS1 has emerged as a promising therapeutic target for neuroinflammation and MDD [33,36]. Previous studies, including those from our group, have demonstrated that inhibiting GLS1 reduces glutamate production in activated microglia and alleviates depression-like behaviors in preclinical models [33,36,37]. Notably, pro-inflammatory cytokines such as TNF-α have been shown to upregulate GLS1 in microglia, while increased GLS1 in turn reinforces microglial inflammatory activation, suggesting a bidirectional mechanism linking cytokine signaling and microglial GLS1 upregulation [31,37,38]. Elevations in glutaminase have also been reported in MDD patients, with significantly increased *GLS1* mRNA levels observed in the postmortem PFC, along with elevated components of neuroinflammatory pathways [38]. Notably, we observed that this upregulated *GLS1* mRNA was also present in microglia, utilizing single-nucleus RNA sequencing (snRNA-seq) data from postmortem brain tissue of MDD patients [39](Fig. 1A), suggesting a role for microglial GLS1 in MDD pathophysiology. However, the use of early GLS1 inhibitors, such as 6-diazo-5-oxo-l-norleucine (DON) [40], has been limited by systemic toxicities, particularly gastrointestinal (GI) side effects, underscoring the need for more selective and safer drug delivery methods [[41], [42], [43]]. Selective allosteric GLS1 inhibitors, such as bis-2-(5-phenylacetamido-1,3,4-thiadiazol-2-yl)ethyl sulfide (BPTES) and CB-839 (Telaglenastat), have mild GI side effects [44], but their poor brain penetration [45] poses a significant limitation to their clinical application for neuroinflammatory diseases such as depression.

To address these limitations, we have utilized a hydroxyl-dendrimer-based delivery system to target activated microglia selectively. Hydroxyl-terminated poly(amidoamine) (PAMAM) dendrimers have demonstrated selective uptake by activated microglia in more than a dozen nervous system disorders and across six species, including primates [[46], [47], [48], [49], [50], [51], [52], [53], [54], [55], [56], [57], [58], [59], [60]]. Notably, the first clinical hydroxyl-dendrimer conjugate showed positive results in a recent Phase II COVID-19 trial (NCT04458298), where it significantly reduced the plasma levels of the neuroinflammatory cytokine Neurofilament light chain (NfL), and improved survival rates in severely impaired hospitalized patients [61]. Given these data, we hypothesized that hydroxyl-dendrimer nanoparticles offer an innovative approach for delivering GLS1 inhibitors directly to microglia, minimizing off-target effects while maximizing therapeutic efficacy. This strategy represents a significant advancement in targeted nanomedicine for treating neuroinflammatory disorders such as MDD.

Building on our previous work, we designed and synthesized two novel GLS1 inhibitor conjugates by attaching structurally distinct GLS1 inhibitors to generation 4 (G4) hydroxyl-terminated PAMAM dendrimers. Specifically, we conjugated the DON analog (9*S*,12*S*,15*S*)-15-amino-9-benzyl-12-(4-diazo-3-oxobutyl)-17-methyl-8,11,14-trioxo-4-oxa-7,10,13-triazaoctadecanoic acid (TTM020) and the BPTES analog *N*-(5-{2-[2-(5-amino- [1,3,4]thiadiazol-2-yl)-ethylsulfanyl]-ethyl}- [1,3,4]thiadiazol-2-yl)-2-phenyl-acetamide (JHU29) [36], resulting in dendrimer-TTM020 (D-TTM020) and dendrimer-JHU29 (D-JHU29) [62], respectively. This study aimed to assess the target engagement and therapeutic efficacy of D-TTM020 and D-JHU29 in a mouse model of chronic social defeat stress (CSDS), a well-established preclinical model of stress-induced depression [37,[63], [64], [65]]. Herein, we report that both D-TTM020 and D-JHU29 inhibited CSDS-elevated GLS1 activity selectively in microglia-enriched CD11b^+^ cells while having no effects on CD11b^−^ cells. From a behavioral perspective, both conjugates alleviated CSDS-induced social avoidance. In addition, D-TTM020 also relieved anxiety-like behaviors and recognition memory deficits caused by CSDS. Importantly, both conjugates were well tolerated and did not cause GI toxicity, which has been reported with previous GLS1 inhibitors [[41], [42], [43], [44]]. This study provides the first demonstration that systemically administered, microglia-targeted dendrimer-conjugated GLS1 inhibitors can selectively inhibit microglial GLS1 activity and improve stress-induced neurobehavioral and cognitive deficits in the CSDS model, while avoiding off-target toxicity commonly associated with conventional GLS1 inhibitors.

## Materials and methods

### Mice

C57BL/6J mice were obtained from Jackson Laboratories (Bar Harbor, ME, USA), and retired CD-1 breeder adults were sourced from Charles River Laboratories (Wilmington, MA, USA). Upon arrival, animals were allowed at least one week of habituation before starting the experiments. Mice were housed under standard conditions, including a 12-h light/dark cycle, at 21 ​°C with 30–70 ​% humidity. All animal procedures were conducted in accordance with protocols approved by the Institutional Animal Care and Use Committee of Johns Hopkins University School of Medicine, ensuring adherence to ethical standards for animal research.

### Murine CSDS model

The CSDS protocol was performed as described previously in male mice [37,63,64,66]. In brief, a test mouse, housed singly, was exposed to a screened, novel aggressive CD-1 mouse (aggressor) for 10 ​min daily over 10 consecutive days. After the 10-min interaction period, the test mouse and the aggressor were separated by a transparent and porous Plexiglas divider, allowing the test mouse to experience chronic psychological stress in the form of a sustained threat for 24 ​h. Non-stressed control mice were housed similarly in separate cages with same-strain companions, which were exchanged daily.

### Materials/reagents

Ethylenediamine-core poly-(amidoamine) [PAMAM] hydroxyl-terminated generation-4 dendrimer [PAMAM G4-OH] was purchased from Dendritech Inc. (Midland, MI). Prior to use, the dendrimer was purified by evaporation, dialyzed using 3 ​kDa MWCO dialysis membranes (Spectrum Laboratories, Rancho Dominguez, CA) and lyophilized, yielding a hygroscopic white solid that was stored at −20 ​°C under argon. Cy5-mono-NHS ester was purchased from Amersham Biosciences-GE Healthcare. All other reagents including 4-(*tert*-butoxycarbonylamino) butyric acid (Boc-GABA-OH), 1-ethyl-3-(3-dimethylaminopropyl)carbodiimide (EDC), 4-dimethylaminopyridine (DMAP), trifluoroacetic acid (TFA), dicyclohexylcarbodiimide (DCC), *N*-hydroxysuccinimide (NHS), *N*,*N*-diisopropylethylamine (DIPEA), benzotriazol-1-yloxytripyrrolidinophosphonium hexafluorophosphate (PyBOP) and anhydrous solvents were purchased from Sigma Aldrich (US).

### Synthesis of D-TTM020

*Synthesis of Fmoc-TTM020:* The synthesis of Fmoc-TTM020 (DON analog for dendrimer conjugation) followed an optimized eight-step process, starting with 3-(2-((*tert* butoxycarbonyl)amino)ethoxy) propanoic acid, and resulting in a total yield of 20 ​%. The steps included esterification with allyl alcohol, Boc deprotection under acidic conditions, amide coupling with Boc-L-Phe-OH and deprotection of Boc. Fmoc-L-DON-OH was next incorporated through second amide coupling, followed by Fmoc deprotection under basic conditions. During the last amide coupling Fmoc-L-Leu-OH was added and in the final step allyl group was deprotected in presence of palladium catalyst Pd(PPh_3_)_4_ and scavenger dimedone, resulting in Fmoc-TTM020. Characterization of Fmoc-TTM020: **^1^H NMR (401 ​MHz, CDCl_3_):** *δ* 0.94, 0.96 (2 ​× ​d, *J* ​= ​6.6, 6H), 1.52–1.77 (m, 3H), 1.88–2.02 (m, 2H), 2.09–2.21 (m, 1H), 2.21–2.36 (m, 1H), 2.60 (t, *J* ​= ​5.8, 2H), 2.87–3.02 (m, 1H), 3.18–3.67 (m, 13 ​H), 3.76 (t, *J* ​= ​5.8, 2H), 4.14–4.34 (m, 3H), 4.36–4.47 (m, 1H), 4.48–4.58 (m, 1H), 4.77–4.89 (m, 1H), 5.25 (bs, 1H), 7.08–7.24 (m, 5H), 7.25–7.35 (m, 2H), 7.35–7.44 (m, 2H), 7.60 (t, *J* ​= ​8.0, 2H), 7.76 (d, *J* ​= ​7.1, 2H).

**^13^C NMR (101 ​MHz, CDCl_3_):** *δ* 21.87, 23.17, 24.87, 27.24, 35.12, 36.18, 38.15, 39.49, 41.51, 47.15, 53.14, 54.05, 54.71, 55.25, 66.73, 67.34, 69.53, 70.26, 70.47, 70.56, 70.59, 120.08, 125.21, 126.68, 127.19, 127.87, 128.43, 129.29, 137.22, 141.33, 141.36, 143.74, 143.89, 156.75, 171.26 (2C), 173.47, 174.48, 195.30. **ESI MS:** 857 (20) ([M+H]^+^), 879 (100) ([M+Na]^+^). **HR ESI MS:** Calcd. for C_45_H_56_O_11_N_6_Na 879.38993. Found 879.39018.

*Synthesis of D-GABA-NH-Boc (****2****):* A solution of PAMAM G4-OH (**1**) (1.00 ​g, 0.07 ​mmol, 1 equiv.) in anhydrous DMF (15 ​mL) was treated with Boc-GABA-OH (0.28 ​g, 1.40 ​mmol, 20 equiv.), DMAP (0.19 ​g, 1.54 ​mmol, 22 equiv.), and the resulting mixture was stirred at room temperature for 5 ​min. Then EDC∗HCl (0.32 ​g, 1.68 ​mmol, 24 equiv.) was added portion-wise over 5 ​min and the reaction mixture was stirred at room temperature for 48 ​h. The crude product was diluted with DMF and was dialyzed against DMF using a 1.0 ​kDa ​MW cut-off cellulose dialysis tubing for 12 ​h, followed by water for 24 ​h. The aqueous solution was lyophilized to yield desired product **2** as a hygroscopic white solid (1.06 ​g, 95 ​%). **^1^H NMR (500 ​MHz, DMSO‑*d*_6_)**: *δ* 1.36 (s, Boc group, 73H), 1.59–1.64 (m, GABA linker-CH_2_, 17H), 2.21 (m, dendrimer CH_2_), 2.45 (m, dendrimer-CH_2_), 2.65–2.73 (m, dendrimer CH_2_), 2.89 (m, dendrimer CH_2_), 3.11 (m, dendrimer-CH_2_), 3.35–3.40 (m, dendrimer CH_2_), 3.39 (t, *J* ​= ​5.0 ​Hz, dendrimer –CH_2_), 3.99 (s, ester linked H, 16H), 4.74 (s, surface OH, 56H), 6.60 (s, GABA amide H, 10H), 7.70–8.10 (m, internal amide H). **HPLC C_18_** (MeCN in H_2_O with 0.1 ​% TFA, linear gradient) retention time: 19 ​min.

*Synthesis of D-GABA-NH*_*2*_ *(****3****):* Compound **3** (500 ​mg, 0.03 ​mmol, 1 equiv.) was dissolved in the mixture of solvents TFA/DCM (3:4) and the reaction mixture was stirred at room temperature for 12 ​h, then diluted with MeOH to remove excess TFA and concentrated *in vacuo*. The crude product was directly used for the next step without further purification. **^1^H NMR (500 ​MHz, DMSO‑*d*_6_)**: *δ* 1.59–1.93 (m, GABA linker-CH_2_), 2.25–3.50 (m, dendrimer-CH_2_), 4.00 (s, ester-linked H), 4.50–5.50 (br s, surface –OH), 7.75–8.50 (m, internal amide H).

*Synthesis of D-GABA-NH-TTM020-NH-Fmoc (****4****):* To the solution of compound **3** (500 ​mg, 0.03 ​mmol, 1 equiv.) in DMF (5 ​mL) were added DCC (61.8 ​mg, 0.30 ​mmol, 10 equiv.) and N-hydroxysuccinimide (34.5 ​mg, 0.30 ​mmol, 10 equiv.) followed by Fmoc-TTM020-COOH (180 ​mg, 0.21 ​mmol, 7 equiv.) and the resulting mixture was stirred at room temperature for 24 ​h. Finally, the mixture was dialyzed against DMF using 1.0 ​kDa for 12 ​h, and against water for additional 24 ​h. The aqueous layer was frozen and lyophilized to obtain the desired product **4** as a pale-yellow product in 75 ​% yield. **^1^H NMR (500 ​MHz, DMSO‑*d*_6_)**: *δ* 0.78–0.91 (TTM isopropyl *H*), 1.13–1.42 (TTM *H*), 1.57–1.74 (TTM *H*), 2.12–2.25 (D-C*H*_2_), 2.27–2.33 (D-C*H*_2_ and TTM *H*), 2.35–2.47 (D-C*H*_2_ and TTM *H*), 2.57–2.71 (TTM *H*), 2.92–3.53 (D-C*H*_2_ and TTM *H*), 3.57 (t, TTM *H*), 4.01 (ester –CH_2_), 4.11–4.51 (TTM020 *H*), 7.14–7.28 (TTM020 Ar*H*), 4.72 (D-O*H*), 7.28–7.73 (Fmoc *H*), 7.75–8.13 (d-amide *H*). **HPLC C_18_** (MeCN in H_2_O with 0.1 ​% TFA, linear gradient) retention time: 30 ​min.

*Synthesis of D-GABA-NH-TTM020-NH2 (D-TTM020,* ***5****):* Compound **4** (100 ​mg, 0.005 ​mmol, 1 equiv.) was dissolved in the mixture of piperidine (1 ​mL) and DMF (4 ​mL) and the resulting mixture was stirred at room temperature for 20 ​min. The reaction mixture was then dialyzed against DMF using 1.0 ​kDa for 12 ​h, followed by dialysis against water for 24 ​h. The aqueous layer was frozen and lyophilized to obtain the desired product **5 (D-TTM020)** as an off-white solid compound in 70 ​% yield. **^1^H NMR (500 ​MHz, DMSO‑*d*_6_)**: *δ* 0.78–0.89 (TTM isopropyl *H*). 1.18–1.42 (TTM *H*), 1.57–1.67 (TTM *H*), 2.11–2.24 (D-C*H*_2_ and TTM *H*), 2.34–2.47 (D-C*H*_2_ and TTM *H*), 2.54–2.84 (TTM *H*), 2.92–3.51 (D-C*H*_2_ and TTM *H*), 3.56 (t, TTM *H*), 3.99 (ester –CH_2_), 4.09–4.29 (TTM020 *H*), 4.39–4.49 (TTM020 *H*), 4.71 (bs, D-O*H*), 7.10–7.27 (TTM020 Ar*H*), 7.72–8.10 (d-amide *H*). **HPLC C_18_** (MeCN in H_2_O with 0.1 ​% TFA, linear gradient) retention time: 10.6 ​min. **MALDI-TOF** observed 18500 ​Da.

### Compound characterization

Nuclear Magnetic Resonance (NMR) spectra were recorded on a Bruker 500 ​MHz spectrometer at ambient temperature, with chemical shifts (ppm) referenced to tetramethylsilane as an internal standard (CDCl3: 1H *δ* 7.27; 13C *δ* 77.0 ​ppm; DMSO‑*d*_6_: 1H *δ* 2.50 ​ppm). Spectra were analyzed using MestReNova software. High-performance liquid chromatography (HPLC) employed a Shimadzu LC-AD system with a Waters BEH300C18 column (5 ​μm, 19 ​× ​250 ​mm), using a linear gradient (0 ​%–90 ​% MeCN in water with 0.1 ​% TFA) at 1.0 ​mL/min, monitored at 210 ​nm via PDA and PDI detectors.

### Drug treatment and experimental schedule

Following 10 consecutive days of CSDS, mice were treated with D-TTM020 (20 ​mg/kg), or D-JHU29 (66.67 ​mg/kg), or vehicle every other day for 14 days using oral gavage. This dosing schedule was selected based on prior studies showing that hydroxyl-terminated PAMAM dendrimers enable sustained delivery to activated microglia, with pharmacodynamic effects lasting at least 48 ​h post-administration [61,67]. One day after the treatment, behavioral characterization was initiated. After all behavioral characterizations were completed, mice were deeply anesthetized with isoflurane, and cardiac perfusions were performed, and the prefrontal cortex (PFC) and hippocampus (HPC) were extracted for cell isolation, and jejunum and colon were harvested for GI histopathology assessments.

### Isolation of microglia-enriched CD11b^+^ cells

Microglia-enriched CD11b^+^ cells were isolated from the PFC and HPC, as we previously described [37]. In brief, brain tissues were dissociated, filtered, and centrifuged. After myelin removal, cells were incubated with CD11b Microbeads (Miltenyi Biotec, Cat # 130-093-634) and separated using LS columns and a quadroMACS magnet. CD11b^+^ cells were collected, washed, and resuspended in HBSS. This antibody-coupled microbead method yields microglia-enriched populations with over 95 ​% purity [68,69]. CD11b^−^ cells were also collected as a control, and both cell types were used for GLS1 activity assessment.

### GLS1 enzyme activity assay

GLS1 enzyme activity was measured in the isolated cell populations as previously described [37]. Briefly, cells were lysed in potassium phosphate buffer (45 ​mM, pH 8.2) with protease inhibitors (Roche, Cat # 04693116001) via sonication. Lysates were incubated with [^3^H]-glutamine (0.09 ​μM, 2.73 ​μCi) for 180 ​min, and the reaction was stopped with imidazole buffer (20 ​mM, pH 7). Unreacted [^3^H]-glutamine was separated using spin columns packed with AG® 1-X2 resin (Bio-Rad, Cat # 140–1251), and [^3^H]-glutamate was eluted and measured using PerkinElmer's TopCount system. Protein concentrations were determined (BioRad, Cat # 5000112), and results were expressed as fmol/mg/h or arbitrary unit (AU).

### Behavioral tests

A series of behavioral tests were performed in the order of open field test (OFT), social interaction test (SIT), novel object recognition test (NORT), and rotarod test.

*OFT:* OFT was performed in a ventilated cabinet using our published protocol [64]. Briefly, mice were placed in the center of an open field arena and allowed to explore for 30 ​min. Locomotor activity was measured using the Photobeam Activity System (PAS–Open Field, San Diego Instruments), which automatically recorded movements, such as ambulation and rearing, through horizontal infrared beam breaks in the x and y axes. The total number of beam breaks was collected over the 30-min period and used as the readout for overall activity.

*SIT:* The three-chamber SIT was conducted as described previously [37,63,64]. Mice were placed in a three-chamber apparatus with equal-sized compartments (each measuring 40 ​cm ​× ​20 ​cm ​× ​26 ​cm) for a three-session trial: 10 ​min habituation, 10 ​min free exploration, and 10 ​min exploration with a conspecific stranger and an inanimate object placed in separate chambers. Mouse behaviors, including movement and sniffing, were recorded using TopScan 3.0 (CleverSys). The apparatus was cleaned with 70 ​% ethanol between trials, and tests were conducted in the dark. The discrimination index was calculated as (time spent sniffing the stranger – time spent sniffing the inanimate object)/(time spent sniffing the stranger ​+ ​time spent sniffing the inanimate object), as described previously [70].

*NORT:* The NORT was conducted following our published protocol with minor modifications [70]. Mice were placed in a Plexiglas open-field arena (20 ​× ​40 ​× ​22 ​cm) with two identical objects secured at adjacent corners. During the 10-min training session, mice were allowed to explore both objects. After 30 ​min, one familiar object was replaced with a novel one, and mice were given 5 ​min to explore. The time spent exploring each object was recorded, and object recognition memory was assessed by calculating the ratio of time spent exploring the novel object to the total exploration time.

*Rotarod Test:* Motor impairment was assessed using a rotarod test as we have previously described [71]. Mice underwent three trials on a 3 ​cm diameter rod (Rotamex 5 rotarod, Columbus Instrument, OH, USA) with settings: Accel 0.006, ACC-IN 005, S-Sp 4.0, and E-Sp 40.0. Mice were habituated on the rod for 1 ​min before the trials. Each trial lasted up to 5 ​min or until the mouse fell. Mice rested for 1 ​min between trials. The maximum speed and latency to fall from each trial were recorded and averaged for analysis.

### Multiplex profiling assay for cytokines in the PFC and HPC

Twenty-four hours after CSDS followed by drug treatment, cytokine and chemokine protein levels in the PFC and HPC were quantified using a multiplex profiling assay platform (Meso Scale Discovery, Rockville, MD, USA), in accordance with the manufacturer's protocol. Briefly, PFC and HPC tissues were collected and homogenized in lysis buffer supplemented with protease inhibitor cocktail (Thermo Fisher, Cat# 87785), then sonicated for protein extraction. After centrifugation (15 ​min at 20,000 ​rpm, 4 ​°C), the soluble fraction was collected. Protein concentrations were determined using the Pierce BCA assay (Thermo Fisher, Cat# A65453), and 105 ​μg protein from PFC or 45 ​μg protein from HPC was used for the MSD Proinflammatory Panel 1 (mouse) Kit (Cat# K15048D).

### Immunofluorescence staining

C57BL/6J mice after 10 days of CSDS (or no CSDS) were injected with dendrimer-Cy5 (D-Cy5 50 ​mg/kg IP) and sacrificed 24h later. Brains were removed, fixed, and cryo-sectioned at a thickness of 20 ​μm for immunofluorescent staining as previously described [71]. In brief, brain sections were permeabilized and blocked using 5 ​% normal goat serum in 1X Tris-buffered saline (TBS) containing 0.1 ​% Triton-X (Millipore Sigma, Cat # 9002-93-1) for 1 ​h at room temperature. After blocking, sections were incubated overnight at 4 ​°C with a primary antibody targeting Iba1(microglia) (Wako, Cat # 019–19471, 1:500) or Aldhl1(astrocyte) (Millipore, Cat # MABN495, 1:100), followed by thorough washing with 1X TBS three times for 5 ​min each. The samples were then treated with secondary antibodies conjugated to Alexa 488 (Invitrogen, Cat # A-11006, 1:400) and Alexa 568 (Invitrogen, Cat # A-11011, 1:400) for 1 ​h at room temperature, followed by the same washing procedure. Then, sections were washed with 1X TBS for 5 ​min three times, mounted with Prolong Glass Antifade Mountant with DAPI (Roche, Cat # 10236276001), and left to dry overnight at room temperature. The slides were stored at 4 ​°C and imaged using a Zeiss LSM 800 confocal microscope (Zeiss, Jena, Germany).

### GI histopathology assessment

Jejunum and colon tissues were harvested from all experimental mouse groups at the conclusion of the study. After fixation in 4 ​% paraformaldehyde (PFA), the samples were transferred to 70 ​% ethanol (one tissue per tube) and submitted to IDEXX BioAnalytics (Columbia, MO, USA) for histopathological assessment. The tissues were processed, including trimming, paraffin embedding, sectioning, and mounting onto slides. Subsequently, hematoxylin and eosin staining was performed, followed by coverslipping and imaging. Histopathological evaluations were conducted based on microscopic alterations. These alterations were graded according to severity using a standardized scale: 0 indicating no significant changes, 1 minimal, 2 mild, 3 moderate, and 4 marked alterations by an observer blinded to the treatment groups. The grading followed the International Harmonization of Nomenclature and Diagnostic (INHAND) criteria (https://www.toxpath.org/inhand.asp). The use of these numerical grades enabled the calculation of a composite lesion score, which facilitated the assessment of both the prevalence and severity of tissue changes within and between groups.

### snRNA-seq data analysis

We leveraged publicly available snRNA-seq data [39] to explore *GLS1* expression in the postmortem PFC tissue of MDD patients (n ​= ​17) and control subjects (n ​= ​17). Raw count matrices were downloaded from NIH GEO repository (GSE144136) and initially processed with ‘Scanpy’ [72]. Potential doublets were detected using ‘Scrublet’ [73] and removed, after which the data were converted to a Seurat object for downstream analyses, including normalization (SCTransform), dimensionality reduction (PCA), clustering, and UMAP visualization [74]. Nuclei with >3 ​% ribosomal content were excluded. Batch effects were corrected using ‘Harmony’ [75].

### Statistical analysis

Statistical analyses were performed by utilizing GraphPad Prism 10. An unpaired Student's *t*-test was used to compare two groups and one-way or two-way ANOVA was used for more than two groups. All quantitative data were presented as mean ​± ​SEM. The significance levels were indicated as ∗∗*p* ​< ​0.01 and ∗ *p* ​< ​0.05.

## Results

### Upregulated GLS1 mRNA in MDD brains was observed in microglia

Significantly increased *GLS1* mRNA levels, along with elevated components of neuroinflammatory pathways, have been reported in the postmortem PFC of patients with MDD [38]. Interestingly, we observed an increase of *GLS1* mRNA in microglia from MDD brains (Fig. 1A). Analysis of snRNA-seq data (GSE144136) from post-mortem dorsolateral PFC (BA9) tissue of MDD subjects who died by suicide [39] showed a strong trend toward a higher proportion of *GLS1*-expressing microglia compared to healthy controls (Fig. 1A–Supplementary Table 1). *GLS1* mRNA upregulation in MDD was also observed in excitatory neurons and oligodendrocytes, whereas no significant changes were detected in astrocytes, endothelial cells, inhibitory neurons, or oligodendrocyte precursor cells (OPCs) (Fig. S1, Supplementary Table 1). These findings support a potential contribution of microglial GLS1 to the pathophysiology of MDD.

### Similar to humans, GLS1 activity was selectively elevated in microglia in CSDS mice

In parallel, to explore whether this observation in humans translates to an animal model, we measured GLS1 activity in microglia-enriched CD11b^+^ cells isolated from the PFC and HPC of CSDS mice (Fig. 1B). GLS1 activity in the CD11b^+^ cells was significantly higher in CSDS mice compared to age-matched controls as we reported previously [37], indicating that chronic stress upregulates GLS1 activity in microglia.

### Synthesis of D-TTM020 and D-JHU029

**Fig. 2:**
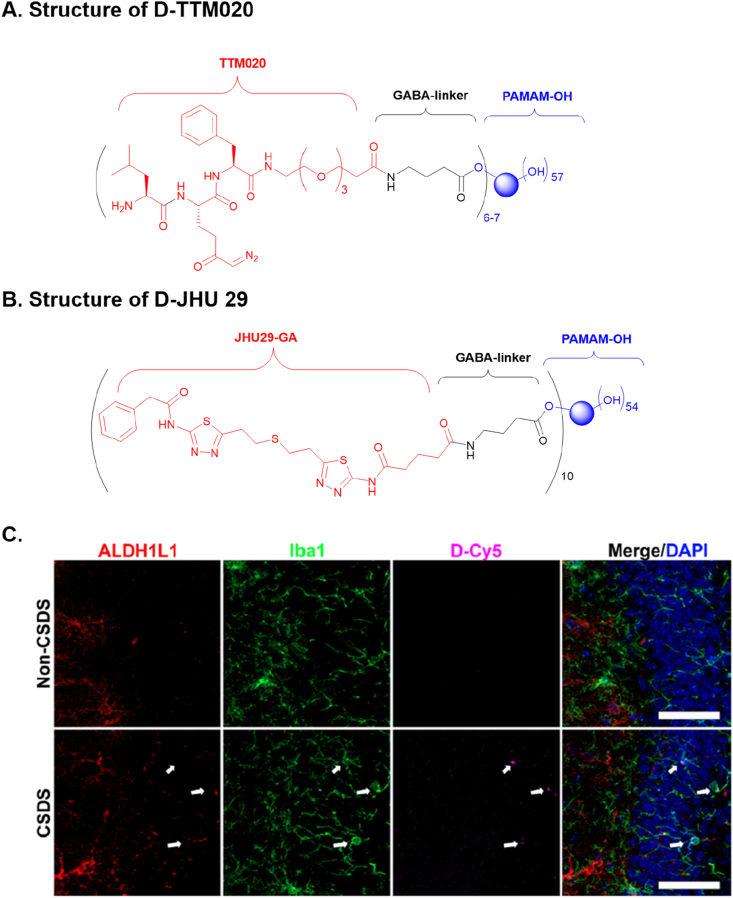
**Structures of dendrimer conjugates and selective uptake of dendrimer-Cy5 (D-Cy5) by microglia in mice after CSDS.** (**A**) Structure of D-TTM020 (dendrimer conjugated TTM020). (**B**) Structures of D-JHU29 (dendrimer conjugated JHU29). (**C**) D-Cy5 was engulfed by microglia in mice after CSDS. Top panel: Non-CSDS mice injected with D-Cy5 did not show fluorescent staining. Bottom panel: CSDS mice had localized Cy5 signal in activated microglia (Iba1), while not in astrocytes (Aldh1l1). White arrows indicated the localization of magenta (D-Cy5), green (Iba1), red (Aldh1l1), blue (DAPI), and merged signals. Scale bar, 50 ​μm.

*Synthesis of D-TTM020*. Structure for dendrimer conjugated TTM020 (D-TTM020) is shown in Fig. 2A. Detailed chemical synthesis and characterization (HPLC and NMR spectra) are shown in Fig. S2. Briefly, PAMAM-G4-OH dendrimer was first modified by reacting with Boc-GABA-OH using EDC and DMAP, followed by Boc deprotection under acidic conditions with TFA. The resulting intermediate containing a primary amine was then reacted to the carboxylic acid group of Fmoc-TTM020, activated using DCC and NHS. The final deprotection of the Fmoc using piperidine led to the formation of D-TTM020 containing 6–7 units of TTM020 per dendrimer representing an approximate 5 ​% of DON loading by weight.

*Synthesis of D-JHU29.* The structure of dendrimer conjugated JHU29-GA (D-JHU29) is shown in Fig. 2B. D-JHU29 was synthesized following our previously published methods [62,76]. In brief, the amine group of JHU29 was reacted with glutaric acid monomethyl ester chloride in the presence of DIPEA to form a reactive handle (JHU29-GA), which was necessary for coupling to the dendrimer. This intermediate was subsequently reacted with G4PAMAM-OH dendrimer, which was pre-modified using Boc-GABA-OH to create a bifunctional dendrimer (G4-NH_2_). The reaction proceeded with PyBOP as the coupling reagent and DIPEA as the base. Final conjugate D-JHU29 was obtained with a loading of 10 molecules of JHU29 per dendrimer, corresponding to an approximate 15 ​% loading by weight. Characterization of D-JHU29 was conducted via ^1^H NMR and HPLC [62].

### Fluorescently labeled hydroxyl-dendrimer (D-Cy5) was selectively engulfed by microglia in mice following CSDS

We synthesized a Cy5 fluorescently-labeled hydroxyl-terminated PAMAM dendrimer (D-Cy5) by partially modifying G4 hydroxyl-terminated PAMAM dendrimers with reactive amine surface end groups, following previously published methods [[77], [78], [79]], to test its brain penetration and microglial targeting in mice subjected to CSDS. After 10 days of CSDS, C57BL/6J mice (or no-CSDS controls) were injected with D-Cy5 (50 ​mg/kg IP) and sacrificed 24 ​h later following transcardial perfusion with PBS. Brains were post-fixed, sectioned, and stained for microglia (Iba1), astrocytes (Aldh1l1), and nuclei (DAPI). In CSDS-exposed mice, activated microglia selectively engulfed the D-Cy5 dendrimer, as indicated by the colocalization of D-Cy5 signals with Iba1-positive microglia near the dentate gyrus of the hippocampus (Fig. 2C, bottom panel). No D-Cy5 signals were observed in non-CSDS control mice (Fig. 2C, top panel). This colocalization confirms the selective uptake of the dendrimer by stress-activated microglia.

### D-TTM020 and D-JHU29 attenuated the elevated GLS1 activity in microglia-enriched CD11b^+^ cells but had no effect on CD11b^−^ cells from CSDS mice

**Fig. 3:**
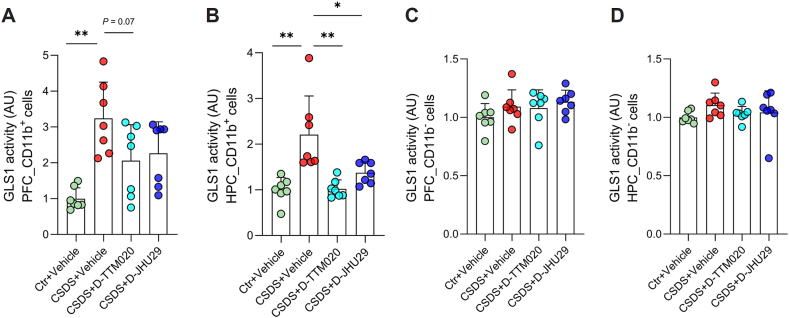
**D-TTM020 and D-JHU29 reduced GLS1 activity selectively in CD11b ​+ cells of mice after CSDS.** (**A**, **B**) GLS1 activity in microglia-enriched CD11b^+^ cells from the PFC (**A**) and HPC (**B**) was significantly elevated in CSDS mice treated with vehicle compared to controls. D-TTM020 tended to inhibit the microglial GLS1 activity increase in PFC, and both D-TTM020 and D-JHU29 treatments significantly reduced GLS1 activity in microglial cells in HPC. (**C**, **D**) GLS1 activity in non-microglial cells from the PFC (**C**) and HPC (**D**) did not show differences between control and CSDS mice or between vehicle and drug-treated groups. Data were presented as mean ​± ​SEM, n ​= ​7 for each group. Statistical significance was determined using one-way ANOVA. ∗∗*p* ​< ​0.01, ∗*p* ​< ​0.05.

The aim of our study was to analyze microglia-targeted GLS1 inhibition as a possible therapeutic approach for chronic stress-associated depression. Thus, we investigated whether D-TTM020 and D-JHU29 could suppress the CSDS-induced upregulation of GLS1 activity in CD11b^+^ (microglia-enriched) and CD11b^−^ (non-microglia) cells. Following CSDS exposure, mice were orally administered D-TTM020 (20 ​mg/kg), D-JHU29 (66.7 ​mg/kg), or a vehicle, and CD11b^+^ and CD11b^−^ cells were isolated from the PFC and HPC 24 ​h post-administration. Protein was extracted from these cells, and GLS1 activity was measured using previously established methods [36,37,69,80]. Consistent with the results shown in Fig. 1B, vehicle-treated CSDS mice exhibited a significant increase in microglial GLS1 activity in both the PFC and HPC, which was normalized by oral administration of D-TTM020 or D-JHU29 (Fig. 3A and B). However, there was no difference in GLS1 activity in non-microglial cells between stressed and control mice, and the administration of D-TTM020 or D-JHU29 did not affect GLS1 activity in non-microglial cells (Fig. 3C and D).

### D-TTM020 and D-JHU29 had no effects on the locomotion of mice after CSDS

**Fig. 4:**
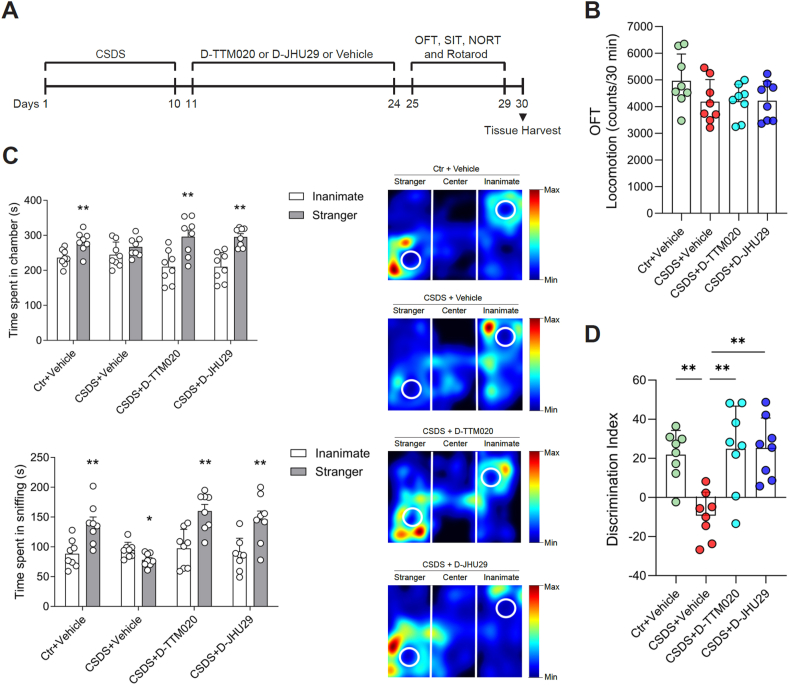
**Effects of D-TTM020 and D-JHU29 on locomotion activity and social avoidance in CSDS-exposed mice.** (**A**) Schematic timeline of the CSDS and treatment protocol followed by behavioral testing. (**B**) Total locomotion in the OFT, measuring general activity levels. (**C**) Time spent in the chambers and time spent sniffing the stranger mouse and inanimate object in the SIT. Heatmaps represented the movement patterns of mice during the test across different treatment groups. (**D**) The discrimination index reflected the preference for the stranger mouse over the inanimate object. Data were presented as mean ​± ​SEM, n ​= ​8 for each group. Statistical significance was determined using one-way ANOVA (**B**, **D**) or paired Student's *t*-test (**C**). ∗*p* ​< ​0.05, ∗∗*p* ​< ​0.01.

To evaluate the effects of D-TTM020 and D-JHU29 on behavioral phenotypes induced by CSDS, we conducted a series of behavioral tests following the CSDS procedure and treatment (Fig. 4A). First, we assessed the locomotion activity of mice using the OFT (Fig. 4B). Locomotion was measured by the total distance traveled in the test, and we found no significant differences across the groups, indicating that neither CSDS nor the treatments affected general locomotion activity (Fig. 4B).

### Both D-TTM020 and D-JHU29 alleviated CSDS-induced social avoidance

Social avoidance is a well-known consequence of CSDS [37,63,64]. To investigate the effects of D-TTM020 and D-JHU29 on CSDS-induced sociability deficits, we employed the three-chambered social interaction test, where mice had the option to interact with an unfamiliar mouse (stranger) or explore an inanimate object (inanimate). As expected, control mice demonstrated a strong preference for the chamber containing the stranger mouse, spending significantly more time in this chamber and engaging in more sniffing behaviors compared to the inanimate object. In contrast, vehicle-treated CSDS mice exhibited social avoidance, showing no significant preference for the stranger mouse chamber and reduced sniffing behavior. Remarkably, treatment with D-TTM020 or D-JHU29 reversed the social avoidance behavior in CSDS-exposed mice. Mice treated with either drug spent significantly more time in the chamber with the stranger mouse and increased their sniffing behavior, indicating a restoration of normal social interaction patterns (Fig. 4C). Heatmaps of the movement patterns during the test show clear spatial preferences, with control and D-TTM020/D-JHU29 treated mice spending more time near the stranger mouse, while vehicle-treated CSDS mice showed little interaction with the stranger mouse chamber (Fig. 4C, heatmaps). Additionally, the discrimination index, which quantifies the preference for the stranger mouse over the inanimate object, was significantly lower in vehicle-treated CSDS mice compared to controls (Fig. 4D). Both D-TTM020 and D-JHU29 treatments significantly increased the discrimination index (Fig. 4D), further confirming their effectiveness in mitigating CSDS-induced social deficits.

### D-TTM020 also relieved CSDS-induced anxiety and recognition memory deficits

**Fig. 5:**
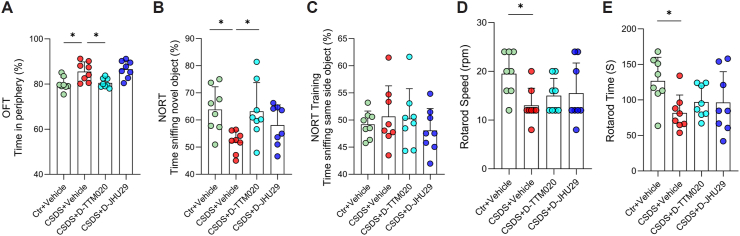
**Effects of D-TTM020 and D-JHU29 on anxiety, recognition memory, and motor coordination in CSDS-exposed mice.** (**A**) Anxiety-like behavior in the OFT was measured as the percentage of time spent in the periphery of the arena. (**B**) Recognition memory in the NORT was quantified as the percentage of time spent exploring the novel object. (**C**) The training phase of the NORT, where the percentage of time spent exploring two identical objects is shown, confirmed no initial object preference. (**D**) Rotarod speed, measuring motor coordination on a rotating rod. (**E**) Rotarod time, assessing motor endurance as the time spent on the rod. Data were presented as mean ​± ​SEM, n ​= ​8 for each group. Statistical significance was determined using one-way ANOVA. ∗*p* ​< ​0.05.

Anxiety-like behaviors and cognitive deficits are well-documented phenotypes in mice subjected to chronic stress [81,82]. Thus, we assessed these behaviors in CSDS-exposed mice to evaluate the potential rescue effects of D-TTM020 and D-JHU29. The anxiety levels of mice were inferred from the percentage of time spent in the periphery during OFT. Vehicle-treated stressed mice spent significantly more time in the periphery compared to age-matched controls, indicating increased anxiety-like behavior. Mice treated with D-TTM020 showed a significant reduction in time spent in the periphery compared to the vehicle-treated CSDS group, indicating that D-TTM020 may have a rescue effect on CSDS-induced anxiety-like behaviors (Fig. 5A).

To further assess the cognitive effects of D-TTM020 and D-JHU29 in CSDS-exposed mice, we conducted the NORT to evaluate recognition memory (Fig. 5B and C). The recognition index was calculated as the percentage of time spent exploring the novel object compared to the total time spent exploring both objects. Vehicle-treated stressed mice showed a significant reduction in the recognition index compared to age-matched controls, indicating impaired recognition memory following CSDS. Mice treated with D-TTM020 demonstrated a significant improvement in the recognition index compared to the vehicle group, suggesting a rescue effect on memory deficits induced by CSDS (Fig. 5B). During the training phase, where mice were exposed to two identical objects, no significant differences in exploration time were observed between the groups, confirming that all mice showed no preference for either object before the test phase (Fig. 5C).

### D-TTM020 and D-JHU29 did not exacerbate the motor deficits caused by CSDS

To evaluate the effects of D-TTM020 and D-JHU29 on motor coordination and balance following CSDS, we performed the rotarod test (Fig. 5D and E). Rotarod speed was measured to assess motor performance (Fig. 5D). Vehicle-treated CSDS mice displayed significantly lower rotarod speeds than control mice, indicating impaired motor coordination due to stress. Although neither D-TTM020 nor D-JHU29 treatment significantly improved rotarod speed compared to the CSDS ​+ ​Vehicle group, both treatments resulted in rotarod speeds that were not statistically different from the control group. This suggests that, while D-TTM020 and D-JHU29 did not restore motor function, they may have prevented further deterioration in motor performance. Rotarod time (Fig. 5E), the time spent on the rotating rod, was also significantly reduced in vehicle-treated CSDS mice compared to controls, reflecting impaired motor endurance. Similar to the rotarod speed, neither D-TTM020 nor D-JHU29 significantly improved rotarod time compared to the CSDS ​+ ​Vehicle group. However, both treated groups exhibited rotarod times that were not statistically different from controls, indicating that while the treatments did not enhance motor endurance, they also did not exacerbate the deficits caused by CSDS.

### D-TTM020 and D-JHU29 reduced CSDS-induced elevation of IL-6 and KC/GRO in the brain

**Fig. 6:**
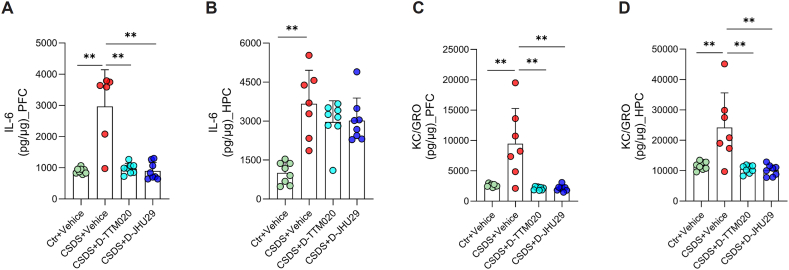
**D-TTM020 and D-JHU29 selectively attenuated CSDS-induced elevation of IL-6 and KC/GRO in the PFC and HPC.** (**A**, **B**) IL-6 protein levels in the PFC (**A**) and HPC (**B**) were measured 24 ​h after CSDS and treatment. (**C**, **D**) KC/GRO protein levels in the PFC (**C**) and HPC (**D**) were measured under the same conditions. Cytokine concentrations were assessed using multiplex assays. Data are presented as mean ​± ​SEM, n ​= ​6–8 per group. Statistical significance was determined using one-way ANOVA. ∗∗*p* ​< ​0.01.

To further characterize the anti-inflammatory effects of D-TTM020 and D-JHU29, we profiled multiple cytokines and chemokines in the PFC and HPC of CSDS-exposed mice using a multiplex assay. CSDS significantly elevated levels of IL-6 and the chemokine KC/GRO in both brain regions compared to controls. Treatment with either D-TTM020 or D-JHU29 markedly reduced IL-6 levels in the PFC, but not in the HPC. Interestingly, KC/GRO levels were significantly reduced by both compounds in both the PFC and HPC (Fig. 6). In contrast, other measured cytokines—including IL-1β, IL-2, IL-5, IL-10, IL-12p70, IFN-γ, and TNF-α—did not show significant changes across groups (Fig. S3), suggesting a selective effect of microglial GLS1 inhibition on key proinflammatory mediators. These findings indicate that D-TTM020 and D-JHU29 attenuate CSDS-induced neuroinflammation without broadly suppressing central immune signaling.

### D-TTM020 and D-JHU29 did not cause GI toxicity in CSDS mice

**Fig. 7:**
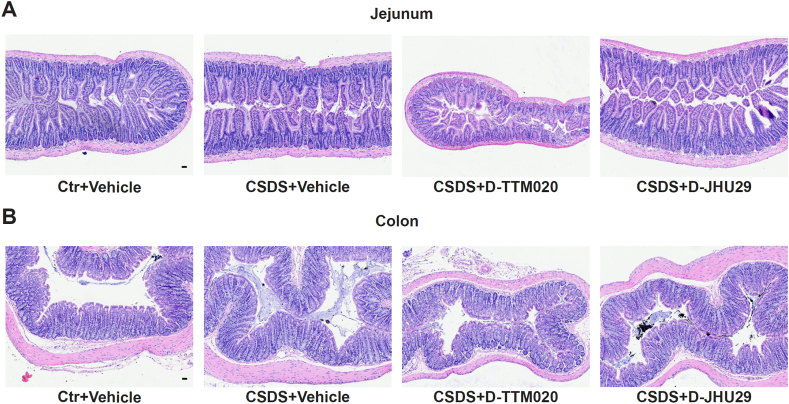
**GI histopathology from D-TTM020- and D-JHU29-treated mice following CSDS.** Representative hematoxylin and eosin-stained sections of the jejunum (**A**) and colon (**B**) from vehicle-treated control mice, vehicle-treated CSDS mice, and CSDS mice treated with D-TTM020 or D-JHU29. Images show the intact mucosal epithelium and the absence of significant pathological changes across all groups. Scale bar, 40 ​μm. Original magnification, 20x.

Previous studies have shown that GLS1 inhibitors like DON were discontinued in clinical development due to systemic toxicities, particularly GI issues such as nausea, gastritis, and diarrhea [[41], [42], [43]], as the GI system is highly dependent on glutamine metabolism [[83], [84], [85]]. To evaluate the potential GI toxicity of D-TTM020 and D-JHU29, jejunum and colon samples were collected from all experimental groups at the end of the study. The tissues were fixed, sectioned, and stained with hematoxylin and eosin for histopathological analysis by IDEXX BioAnalytics. Microscopic evaluation revealed no significant differences in the microanatomic structure of the jejunum (Fig. 7A) or colon (Fig. 7B) across all groups, including vehicle-treated controls, vehicle-treated CSDS mice, and those CSDS mice treated with D-TTM020 or D-JHU29. All structures were within normal histological limits, with no signs of inflammation or tissue damage. These observations were further supported by the histopathological scoring data (Table 1, Table 2), where no significant abnormalities or lesions were identified in any of the treatment groups. These results suggest that neither D-TTM020 nor D-JHU29 induced GI-related toxicities in stressed mice.

**Table 1:** Jejunum histopathological findings∗.

	Ctr ​+ ​Vehicle			CSDS ​+ ​Vehicle			CSDS ​+ ​D-TTM020			CSDS ​+ ​D-JHU29		
	# Abnormal	Mean Group Score	Mean Lesion Score	# Abnormal	Mean Group Score	Mean Lesion Score	# Abnormal	Mean Group Score	Mean Lesion Score	# Abnormal	Mean Group Score	Mean Lesion Score
Significant findings identified	0	0.0		0	0.0		0	0.0		0	0.0	
Artifact	0	0.0		0	0.0		0	0.0		0	0.0	
Sum - scores	0	0.0	0.0	0	0.0	0.0	0	0.0	0.0	0	0.0	0.0
No significant finding samples	3			3			3			3		

∗ Microscopic changes were graded, as to severity, utilizing a standard grading system whereby 0 = no significant change, 1 = minimal, 2 = mild, 3 = moderate, and 4 = marked. International Harmonization of Nomenclature and Diagnostic (INHAND) Criteria standards are used as the basis of evaluation (https://www.toxpath.org/inhand.asp). N = 3 mice for each group.

**Table 2:** Colon histopathological findings^a^.

	Ctr ​+ ​Vehicle			CSDS ​+ ​Vehicle			CSDS ​+ ​D-TTM020			CSDS ​+ ​D-JHU29		
	# Abnormal	Mean Group Score	Mean Lesion Score	# Abnormal	Mean Group Score	Mean Lesion Score	# Abnormal	Mean Group Score	Mean Lesion Score	# Abnormal	Mean Group Score	Mean Lesion Score
Significant findings identified	0	0.0		0	0.0		0	0.0		0	0.0	
Artifact	0	0.0		0	0.0		0	0.0		0	0.0	
Sum - scores	0	0.0	0.0	0	0.0	0.0	0	0.0	0.0	0	0.0	0.0
No significant finding samples	3			3			3			3		

a Microscopic changes were graded, as to severity, utilizing a standard grading system whereby 0 ​= ​no significant change, 1 ​= ​minimal, 2 ​= ​mild, 3 ​= ​moderate, and 4 ​= ​marked. International Harmonization of Nomenclature and Diagnostic (INHAND) Criteria standards are used as the basis of evaluation (https://www.toxpath.org/inhand.asp). N ​= ​3 mice for each group.

## Discussion

In this study, we identified microglial GLS1 as a novel target for depression treatment, supported by both clinical and preclinical evidence. Dysregulated glutamate signaling has been increasingly recognized as a critical factor in the pathophysiology of MDD [[9], [10], [11],^13^,^15^]. Specifically, GLS1 plays a key role in brain glutamate production [[31], [32], [33],^36^] and has been found to be significantly upregulated in the postmortem PFC of MDD patients [38]. Notably, we found that *GLS1* mRNA upregulation is also observed in PFC microglia of MDD patients (Fig. 1A), though not restricted to this cell type (Fig. S1). Our preclinical data are complementary to these human findings, showing a pronounced and selective increase in microglial GLS1 activity in the CSDS mouse model. While *GLS1* mRNA expression was not measured in this study, our previous work demonstrated that GLS1 activity was elevated in microglia following CSDS without a corresponding increase in microglial mRNA levels, suggesting post-transcriptional regulation [37]. Importantly, we previously demonstrated that the brain-penetrable GLS1 inhibitor JHU083, a prodrug of DON, was able to normalize this microglial-specific increase in GLS1 activity and showed significant efficacy in alleviating CSDS-induced psychosocial behavior deficits [37]. These results establish that GLS1 is not only elevated in the context of depression but also that inhibiting GLS1 activity can reverse depressive-like behaviors, positioning GLS1 as a promising therapeutic target for MDD.

Despite the promising results obtained with JHU083, its translational potential is limited due to GI toxicity, a well-known side effect of DON [[41], [42], [43]]. JHU083 was specifically designed to enhance brain penetration and reduce peripheral toxicity, but its safety profile remains insufficient for long-term clinical use. Therefore, we explored a more innovative approach by conjugating two structurally distinct GLS1 inhibitors, TTM020 (DON analog) and JHU29 (BPTES analog) (Fig. 2A–B), to G4 hydroxyl-terminated PAMAM dendrimers, which are known to specifically target activated microglia. Dendrimer-conjugated nanoparticles have emerged as a cutting-edge therapeutic strategy, enabling precise drug delivery to specific cell populations. Hydroxyl-dendrimer-based approaches have shown microglial/macrophage targeting and therapeutic efficacy in various animal models, including those of neuroinflammation and CNS diseases [47,[51], [52], [53], [54], [55], [56],^58^,^62^,^86^]. Furthermore, our recent clinical data demonstrated that a similar dendrimer conjugate was well tolerated with no significant long-term safety concerns, and led to reduced neuroinflammatory cytokine NfL, improved neurological biomarkers, and increased survival in patients with severe COVID-19 [61]. This evidence supports the safety and efficacy of the dendrimer delivery system and highlights its translational potential.

In this study, both D-TTM020 and D-JHU29 showed significant improvements in CSDS-induced social avoidance (Fig. 4C and D). These effects are consistent with prior reports showing that microglial GLS1 upregulation contributes to neuroinflammation and glutamate dysregulation in models of stress and neuroinflammatory conditions [37,65]. Moreover, the hydroxyl-dendrimer-GLS1 inhibitor conjugates demonstrated a favorable safety profile, with no observable neurological deficits or GI side effects, a critical improvement over previous broad-spectrum and selective GLS1 inhibitors (Fig. 5, Fig. 6). These results underscore the potential of GLS1 inhibition as a novel therapeutic strategy for depression, particularly when delivered in a microglia-targeted manner.

The rationale for targeting microglial GLS1 is rooted in the growing recognition of the role of microglia in the pathophysiology of depression. Microglia, the brain's resident immune cells, are key mediators of neuroinflammation, and their dysregulation has been implicated in the development of depression and other neuropsychiatric disorders [38,[87], [88], [89], [90], [91], [92]]. In our study, we assessed microglia in CSDS-exposed mice using Iba1 immunostaining and found that Cy5-labeled hydroxyl-dendrimers were selectively taken up by Iba1^+^ microglia (Fig. 2C). Given that CSDS has been shown to induce robust microglial activation [93,94], and that our previous work demonstrated hydroxyl-terminated PAMAM dendrimers are preferentially internalized by activated microglia and accumulate in lysosomes via fluid-phase endocytosis [57,60,95], the observed uptake supports the interpretation that the dendrimers were taken up by activated microglia in this model. Once internalized, the GLS1 inhibitor is gradually released from the dendrimer within the lysosomal environment, allowing it to diffuse locally and inhibit GLS1 activity in the cytosol or at the mitochondrial periphery, as supported by prior in vitro release data under lysosomal-mimicking conditions [62,96]. For quantitative assessment, our recent study further confirmed selective microglial uptake using immunofluorescence and flow cytometry, with ∼17.5-fold and ∼10.6-fold higher Cy5 signal in microglia relative to astrocytes and neurons, respectively [71]. This selective uptake pattern was not observed in non-CSDS controls, likely due to the absence of microglial activation and the limited permeability of the blood-brain barrier (BBB) under baseline conditions. Previous studies have shown that hydroxyl-terminated PAMAM dendrimers are not internalized by resting microglia or other brain cells in healthy animals [47,57,60,71,96]. Additionally, while CSDS does not cause gross BBB disruption, it may lead to subtle, region-specific increases in BBB permeability [97], potentially facilitating localized dendrimer access to stress-affected brain regions.

Recent studies, including ours, have shown that microglia-driven neuroinflammatory processes have been linked to depressive-like behaviors in preclinical models, including social withdrawal and anhedonia [37,65]. To further evaluate this mechanism, we measured cytokine levels in the PFC and HPC of CSDS-exposed mice and found that both D-TTM020 and D-JHU29 significantly reduced the CSDS-induced upregulation of proinflammatory mediators, including the cytokine IL-6 and the chemokine KC/GRO (Fig. 6). These effects are consistent with our previous finding that GLS1 inhibition in microglia-enriched CD11b^+^ cells suppress the CSDS-induced increase in mRNA expression of proinflammatory cytokines [37]. Mechanistically, these benefits may be mediated by suppression of glutamate overproduction and disruption of proinflammatory metabolic reprogramming in activated microglia, thereby limiting neuroinflammatory signaling and excitotoxicity [29,[98], [99], [100]]. By selectively inhibiting microglial GLS1, our approach addresses a central mechanism underlying the neuroinflammatory component of depression, offering a targeted and innovative therapeutic strategy.

Interestingly, while both D-TTM020 and D-JHU29 exhibited significant efficacy in alleviating CSDS-induced social avoidance, we observed that D-TTM020 also relieved CSDS-induced anxiety-like behaviors and recognition memory deficits. These therapeutic differences may be attributed to their distinct chemical structures, leading to variations in their pharmacokinetics and pharmacological mechanisms. As D-TTM020 releases a covalent and broad-spectrum inhibitor (Fig. 2A), it may suppress microglial GLS1 more effectively and/or for a longer duration (Fig. 3). Additionally, its enhanced efficacy may stem from its ability to inhibit multiple glutamine-utilizing enzymes beyond GLS1 [98,101]. These possibilities, along with potential pharmacokinetic differences between the two conjugates, may underlie their differential behavioral effects and merit further investigation. Understanding these mechanisms will be critical for optimizing the clinical use of these compounds and ensuring maximal therapeutic benefits in depression and related disorders.

One limitation of this study is that we conducted experiments exclusively on male mice. We acknowledge the importance of including female mice to fully understand the therapeutic potential of hydroxyl-dendrimer-GLS1 inhibitor conjugates, as sex differences can influence the pathophysiology and treatment outcomes of depression. Our laboratory has already established the CSDS paradigm for female mice. However, to minimize the use of animals, we initially focused on male mice to explore the efficacy of the two conjugates as a proof-of-concept study. Based on the optimized conjugates identified in the current research, future studies will be expanded to include female mice to further validate and extend our findings. Another limitation is that we did not stratify CSDS-exposed mice into susceptible and resilient subgroups. Given the known heterogeneity in behavioral responses to chronic stress, future studies will include such stratification to determine whether microglial GLS1 activity differs between these subpopulations. Moreover, the impact of GLS1 inhibition on physiological microglial functions such as synaptic pruning and phagocytosis remains uninvestigated and warrants further investigation. Finally, while mouse data demonstrate a role of microglia-specific GLS1 activity increase in depressive behavior, in humans *GLS1* mRNA is increased not only in microglia but also in excitatory neurons and oligodendrocytes. This suggests that further studies are needed to disentangle the cell-specific contributions of GLS1 to MDD and to explore possible cross-talk between microglia, neurons, and oligodendrocytes in modulating glutamate metabolism.

Building on our current findings with G4 hydroxyl-dendrimer-GLS1 inhibitor conjugates, future work could involve developing and evaluating G6 hydroxyl-dendrimer-GLS1 inhibitor conjugates to compare efficacy and safety. Previous studies have demonstrated that G6 hydroxyl-terminated PAMAM dendrimers exhibit extended plasma circulation time and enhanced tissue exposure in activated regions [58,102,103], albeit with potential limitations in CNS transport due to increased size. By assessing both G4 and G6 conjugates, we aim to identify the optimal dendrimer generation for microglial GLS1 targeting, which could further enhance the therapeutic window and CNS retention of GLS1 inhibitors in stressed models.

In conclusion, our findings provide strong support for the therapeutic potential of microglial GLS1 inhibition in depression. The development of hydroxyl-dendrimer-GLS1 inhibitor conjugates, D-TTM020 and D-JHU29, represents a significant advancement in targeted depression treatment, offering the potential for improved efficacy and safety compared to traditional therapies. Our approach not only addresses the neuroinflammatory component of depression but also minimizes systemic toxicity, making it a promising candidate for clinical translation. Future studies will focus on optimizing the pharmacological properties of these compounds and evaluating their efficacy in clinical trials, with the goal of providing more targeted and effective treatments for patients with chronic stress-associated depression.

## Author contributions

M.H. and Y.L. performed cellular, behavioral, immunohistochemical, and histopathological experiments and contributed to data analysis. J.H. and G.U. conducted post-mortem MDD brain snRNAseq analysis in collaboration with X.Z. A.S., W.L., T. Tichý, L.T., N·H., P.M., R.M.K., T. Tsukamoto, and R.R. designed, synthesized, and validated D-TTM020 and D-JHU29 together with B·S.S. A.G.T. performed GLS1 activity assays and related analyses. Y.S. and M.O. contributed to behavioral and histopathological experiments alongside M.H. and Y.L. B.S.S. and X.Z. conceptualized the study and drafted the manuscript, with contributions from M.H. and Y.L. All authors reviewed and approved the final manuscript.

## Data availability

Data is available on request from the corresponding authors.

## Declaration of competing interest

The authors declare the following financial interests/personal relationships which may be considered as potential competing interests: Xiaolei Zhu reports financial support was provided by National Institutes of Health. Barbara S. Slusher reports financial support was provided by National Institutes of Health. Xiaolei Zhu reports a relationship with National Institutes of Health that includes: funding grants. Barbara S. Slusher reports a relationship with National Institutes of Health that includes: funding grants. Authors Barbara S. Slusher, Xiaolei Zhu, Rana Rais, Rangaramanujam M. Kannan, Anjali Sharma, Takashi Tsukamoto, Pavel Majer, Tomáš Tichý, and Lukáš Tenora are listed as inventors in patent applications filed by Johns Hopkins Technology Ventures covering novel compositions of glutamine antagonists and their dendrimer conjugates. This arrangement has been reviewed and approved by the Johns Hopkins University in accordance with its conflict of interest policies. The authors declare no competing interests. If there are other authors, they declare that they have no known competing financial interests or personal relationships that could have appeared to influence the work reported in this paper.

## Figures and Tables

**figs1:**