# Supplementary material for: Inhibition of microglial glutaminase alleviates chronic stress-induced neurobehavioral and cognitive deficits
Source: Neurotherapeutics. 2025 Sep 27;22(6):e00759. doi: 10.1016/j.neurot.2025.e00759 (PMC12664560; doi:10.1016/j.neurot.2025.e00759)
Supplement: Multimedia component 1 [file mmc1.docx]

# Supplementary information

Inhibition of microglial glutaminase alleviates chronic stress-induced neurobehavioral and cognitive deficits

# Meixiang Huang^a,b,1^, Yannan Li^c,1^, Ajit G. Thomas^a^, Anjali Sharma^d^, Wathsala Liyanage^d^, Tomáš Tichý^e^, Lukáš Tenora^a,e^, Yu Su^a,b^, Jisu Ha^f^, Niyada Hin^a^, Mizuho Obayashi^c^, Pavel Majer^e^, Rangaramanujam M. Kannan^d,g^, Takashi Tsukamoto^a,b,h^, Gianluca Ursini^c,f^, Rana Rais^a,b,h^, Barbara S. Slusher^a,b,c,h,i,j,k,*^, Xiaolei Zhu^a,c,*^

^a^Johns Hopkins Drug Discovery, Johns Hopkins University School of Medicine, Baltimore, MD 21205, USA

^b^Department of Neurology, Johns Hopkins University School of Medicine, Baltimore, MD 21205, USA

^c^Department of Psychiatry and Behavioral Sciences, Johns Hopkins University School of Medicine, Baltimore, MD 21205, USA

^d^Center for Nanomedicine, Department of Ophthalmology, Wilmer Eye Institute, Johns Hopkins University School of Medicine, Baltimore, MD 21231, USA

^e^Institute of Organic Chemistry and Biochemistry, Academy of Sciences of the Czech Republic (ASCR), Prague 160 00, Czech Republic

^f^Lieber Institute for Brain Development, Johns Hopkins Medical Campus, Baltimore, MD 21205, USA

^g^Department of Chemical and Biomolecular Engineering, ^h^Department of Physiology, Pharmacology & Therapeutics, ^i^Department of Neuroscience, ^j^Department of Oncology, ^k^Department of Medicine, Johns Hopkins University School of Medicine, Baltimore, MD 21205, USA

^1^These authors equally contributed to this work and are designated as co-first authors.

^*^Corresponding authors. E-mail addresses: bslusher@jhmi.edu (B.S.S), xzhu31@jhmi.edu (X.Z).

Running title: Microglial Glutaminase Inhibition in Stress.

**
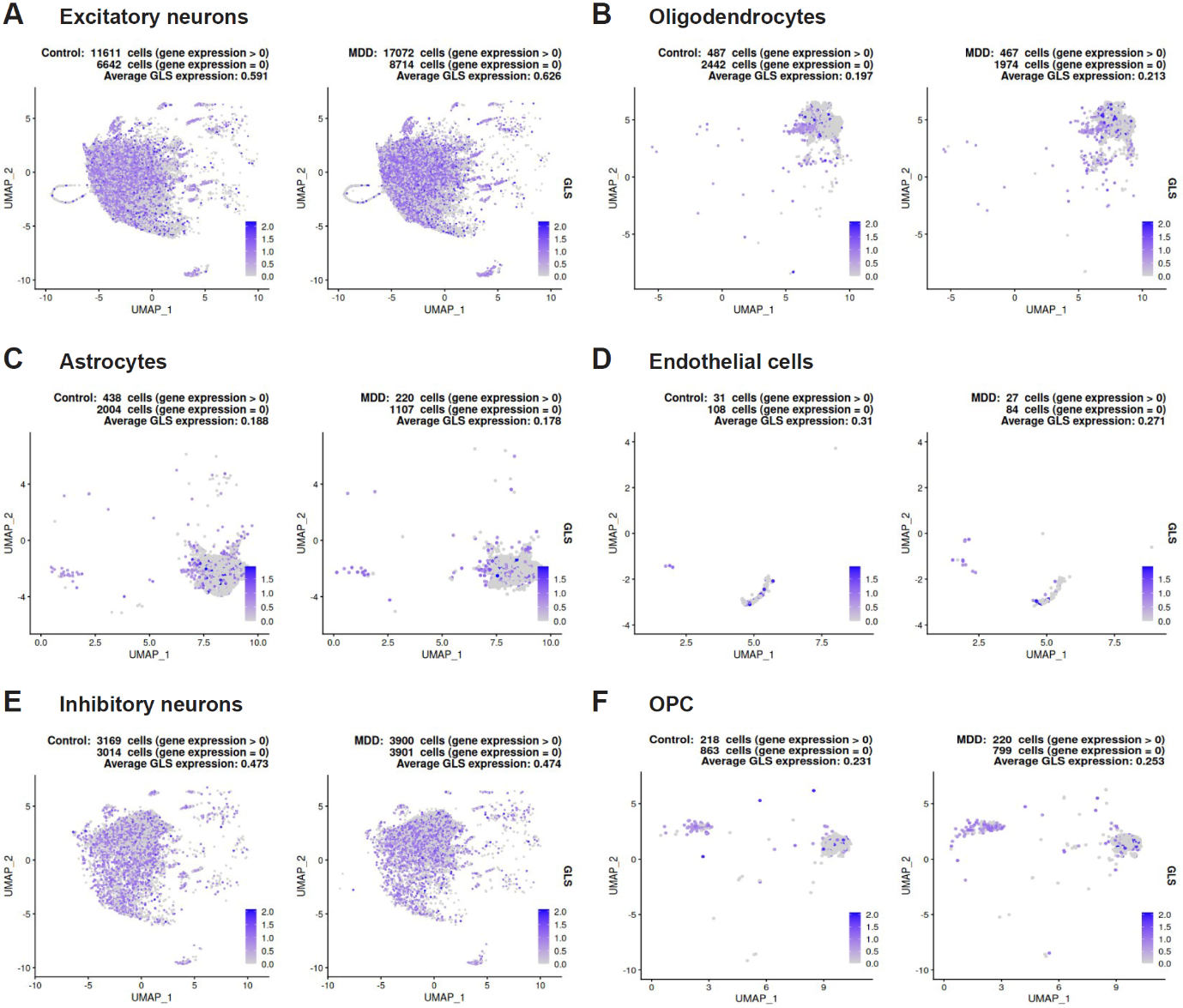
**

**Fig. S1.** ***GLS1* expression at the single-nucleus level in post-mortem MDD cases.** The Uniform Manifold Approximation and Projection (UMAP) plot colored by velvet showing nuclei expressing *GLS1* mRNA in MDD cases (right) vs. control subjects (left), in (**A**) excitatory neurons, (**B**) oligodendrocytes, (**C**) astrocytes, (**D**) endothelial cells, (**E**) inhibitory neurons, and (**F**) oligodendrocyte precursor cells (OPC). Control subjects, n = 17, MDD cases, n = 17. Data were analyzed by χ^2^ tests. See Supplementary Table 1 for statistics and further details.

**
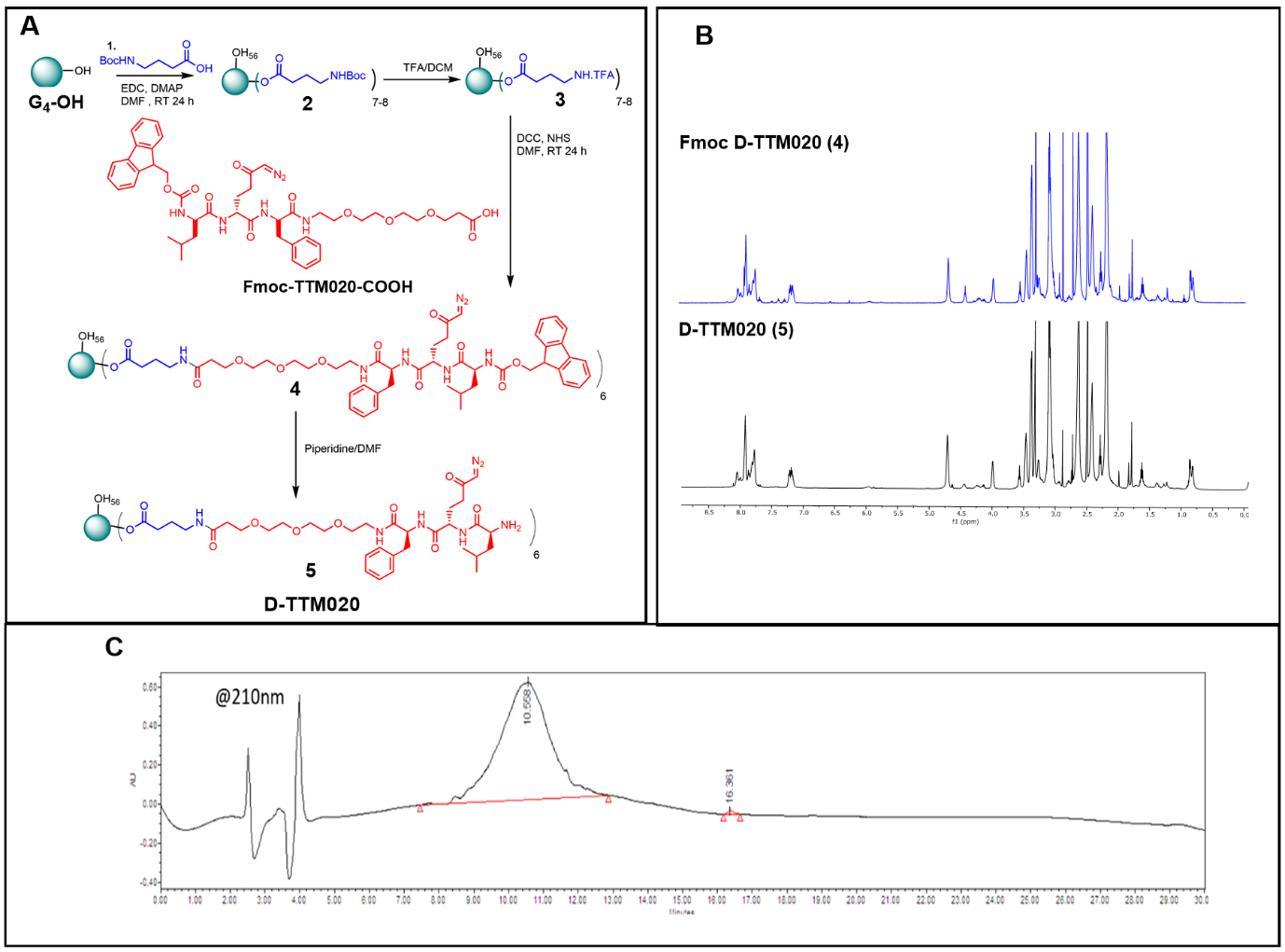
**

**Fig. S2.** (**A**) Schematic representation of the synthesis of D-TTM020. (**B**) ^1^H NMR traces of Fmoc TTM020 and D-TTM020. (**C**) HPLC traces of D-TTM020 at 210 nm.


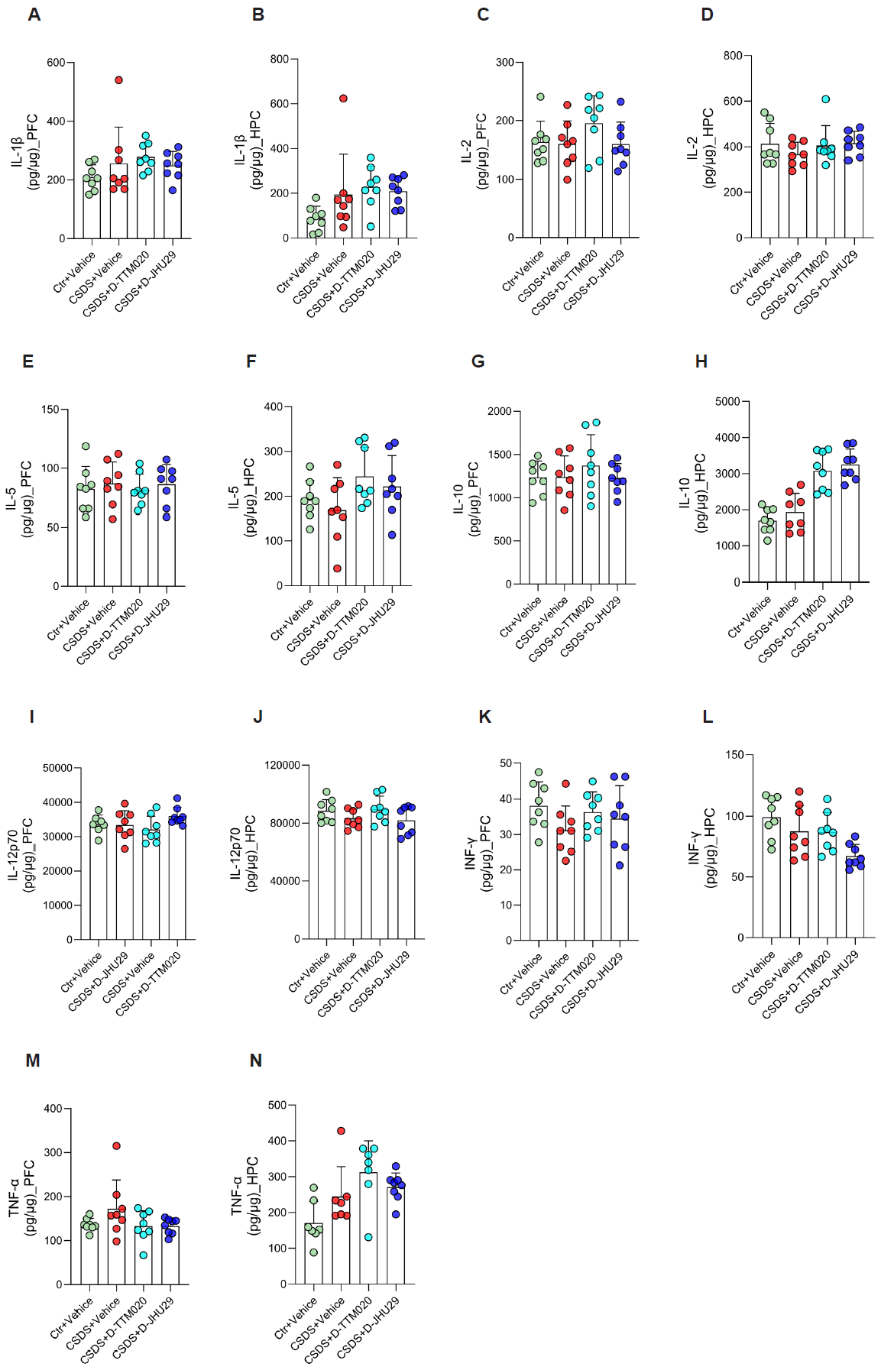


**Fig. S3. Effects of D-TTM020 and D-JHU29 on other cytokines in the PFC and HPC after CSDS.** (**A**–**N**) Protein levels of IL-1β, IL-2, IL-5, IL-10, IL-12p70, IFN-γ, and TNF-α in the PFC and HPC were measured 24 hours after CSDS and treatment. Cytokine concentrations were assessed using multiplex assays. Data are presented as mean ± SEM, n = 6–8 per group. Statistical significance was determined using one-way ANOVA.
